# Genomic Features of the Micropredator *Lysobacter* sp. Hz25 Isolated from the Rhizosphere of *Hedysarum zundukii*

**DOI:** 10.3390/ijms27093800

**Published:** 2026-04-24

**Authors:** Ivan S. Petrushin, Yulia V. Nurminskaya, Yulia A. Markova

**Affiliations:** Siberian Institute of Plant Physiology and Biochemistry, Siberian Branch of the Russian Academy of Sciences, Irkutsk 664033, Russia; julosti@yandex.ru (Y.V.N.); juliam06@mail.ru (Y.A.M.)

**Keywords:** *Lysobacter antibioticus*, comparative genomics, chitinase, secretion system, lytic enzymes, type III secretion system, arsenic resistance, micropredator, rhizosphere, Lake Baikal

## Abstract

*Lysobacter antibioticus* Hz25 is a novel strain that was isolated from the rhizosphere of the relict endemic plant *Hedysarum zundukii* Peschkova (Fabaceae), which grows on carbonate soils in the Baikal region of Russia. This work presents the complete genome sequence of Hz25 (5.98 Mb, 66.94% GC), which was obtained using a hybrid assembly method combining Oxford Nanopore and Illumina sequencing. Phylogenetic analysis based on 47 *Lysobacter* genomes and an average nucleotide identity (ANI) value of 96% confirmed its affiliation with *L. antibioticus*. A comparative pan-genome analysis with three closely related strains (13-6, 76, and ATCC 29479) identified 554 strain-specific genes. This significant genomic plasticity likely reflects adaptation to the sharply continental climate, high insolation, and low free iron content of the native soil. The genome encodes a comprehensive micropredator arsenal, including: seven chitinase genes (GH18 and GH19 families); bacteriolytic enzymes (Blp, L1, L4, Ami); a complete type III secretion system (T3SS) with predicted effectors; type IV pili (including the PilZ-PilB regulatory complex); and siderophore biosynthesis genes (lysochelin). The genome contains genes *ars* of an arsenic resistance system, but lacks the ACR3 efflux pump, suggesting that these genes may have alternative functions. Genes involved in calcium homeostasis (Excalibur domain, Na^+^/Ca^2+^ antiporter) were also identified. These features make Hz25 a promising candidate for biocontrol applications in cold climates and metal-contaminated environments.

## 1. Introduction

The genus *Lysobacter* was first described by P. Christensen and F. D. Cook in 1978 [1]. Currently, more than 90 species are recognized within this genus (https://lpsn.dsmz.de/genus/lysobacter, accessed on 31 March 2026). Bacteria belonging to the genus *Lysobacter* (from the Xanthomonadaceae complex) are Gram-negative rods capable of synthesizing a wide range of active substances that are effective against Gram-negative and Gram-positive bacteria, fungal pathogens, oomycetes, nematodes, and insects [2]. Members of the genus are generally characterized by a high G+C content (61–70%) and a genome size of 3–6 Mb according to NCBI GenBank (https://www.ncbi.nlm.nih.gov/datasets/genome/?taxon=68, accessed on 30 March 2026). Twitching motility, mediated by type IV pili (T4P), enables these bacteria to successfully adopt a micropredatory lifestyle [3].

*Lysobacter* employs a variety of strategies to attack bacterial and fungal cells. The genus is characterized by the secretion of bioactive molecules. These include antibiotics from various classes, such as the heat-stable antifungal factor (HSAF), phenazine derivatives (e.g., myxin), pyrrole-2-carboxylic acid, diketopiperazines (e.g., cyclo(L-Pro-L-Tyr)), and cyclic lipodepsipeptides (e.g., lysobactin and lysocin), among others [2,4,5]. *Lysobacter* also secretes a diverse array of lytic enzymes, including proteases, amidases, glucanases, and chitinases [2]. Many of these micropredatory strategies require direct contact with the target cell. For instance, the type IV secretion system (T4SS), described in *L. enzymogenes* OH11, allows for the direct injection of effector proteins into the target cell [6]. In addition to T4SS, the genomes of *Lysobacter* species contain sequences that encode secretion systems of types I, II, III, IV, V, and VI [2]. Furthermore, *Lysobacter* utilizes outer membrane vesicles (OMVs) to deliver bioactive metabolites, a mechanism observed in *L. enzymogenes* C3 and OH11, as well as in *L. capsici* XL1 [7,8].

Because iron is a limiting resource in soil environments, *Lysobacter* secretes siderophores. Accordingly, genes involved in lysochelin and other siderophore biosynthesis are frequently found in their genomes [2]. A characteristic feature of *Lysobacter* is its resistance to arsenic [9] and heavy metals such as copper [10], lead [11], and others. The diversity of *Lysobacter* species has attracted considerable attention as a valuable source of novel biocontrol agents [2].

Among the species within the genus, *Lysobacter antibioticus* is particularly known for the biosynthesis of phenazine antibiotics [12], notably the di-N-oxide phenazine myxin, which is active against various phytopathogenic bacteria [13,14]. Six different phenazines have been identified in *L. antibioticus* [15], and this valuable trait has even been transferred to the more closely related species *L. enzymogenes* using genetic tools [16]. Several *L. antibioticus* strains have been analyzed in the literature, including 13-6, HS124, OH13, and ATCC 29479. Studies on strain OH13 have elucidated myxin biosynthesis, including the characterization of the O-methyltransferase enzyme LaPhzM and the investigation of self-protection mechanisms against its own antibiotic, which are mediated by the LexABC efflux pump [12,17,18]. Strain 13-6 is notable for its ability to increase indole-3-acetic acid (IAA) production to a level of up to 63.85 μg/mL, and to solubilize phosphate. Moreover, phosphate solubilization genes were discovered in this strain for the first time in the genus *Lysobacter* [19]. Strain HS124 demonstrates activity in the biological control of fungi, nematodes, and insects through the production of chitinases, proteases, and a novel antifungal compound [20,21]. Strain ATCC 29479 was found to harbor a silent biosynthetic gene cluster that produces the anti-MRSA cyclic lipodepsipeptide WBP-29479A1 [22]. Nevertheless, comparative genomic analyses within the *L. antibioticus* species remain relatively rare. This is particularly true for strains isolated from unique ecological niches.

Genomic analysis of new strains, particularly those isolated from unique ecotopes, can reveal the genetic basis for the distinctive regulatory and ecological roles of *Lysobacter*. Currently, only four complete genomes of this species are available (https://www.ncbi.nlm.nih.gov/datasets/genome/?taxon=84531, accessed on 31 March 2026).

This study aims to determine the taxonomic position of Hz25 and describe its genomic features. This strain was isolated from the rhizosphere of the relict endemic plant *Hedysarum zundukii* Peschkova (Fabaceae), which grows on carbonate soils in the Baikal region. These soils are characterized by an alkaline pH (8.7), low availability of iron and phosphorus, and high calcium content, creating extreme conditions that likely influence the adaptive traits of the rhizosphere microbial community. Some of its features have been confirmed experimentally, including pili-based gliding and micropredatory behavior, which is manifested as the cooperative gliding of cells towards a prey microorganism using type IV pili [23]. Investigating the genomic features of Hz25 will guide future experimental work to unlock the biotechnological potential of this strain.

## 2. Results

### 2.1. Cultivation of Cells, Genome Assembly, Scaffolding and Features

The strain was isolated from the rhizosphere of *Hedysarum zundukii* Peschkova (Fabaceae), a narrow-local endemic of the Western coast of Lake Baikal. The bacteria were well cultivated using SYM medium (sucrose, 10 g/L; yeast extract, 5 g/L) containing agar (8 g/L) [24].

The genome was sequenced using the Oxford Nanopore (ONT; MinION; Flow Cell R10.4.1, Oxford, UK) and Illumina NovaSeq 6000 (2 × 100 bp, San Diego, CA, USA) platforms. The raw data were filtered by fastp (Illumina reads) and by filtlong (ONT reads). Hybrid assembly was performed using SPAdes v. 3.13.1 and has 5,985,541 bp length with GC % = 66.94. The final genome has been released in NCBI GenBank (acc. number CP175666, https://ncbi.nlm.nih.gov/nuccore/CP175666, accessed on 1 December 2024) for further study and annotation. Assembly statistics are presented in Table 1. Genome completeness analysis with benchmarking universal single-copy orthologs (BUSCO v. 5.8.0) using “xanthomonadales_odb10” database, mode “genome” and other settings set to default showed that Hz25 genome has 98.9% complete, 0.3% fragmented, and 0.8% missing of 366 total BUSCO groups.

### 2.2. Phylogenetic Relationship with Closer Species

**Figure 1:**
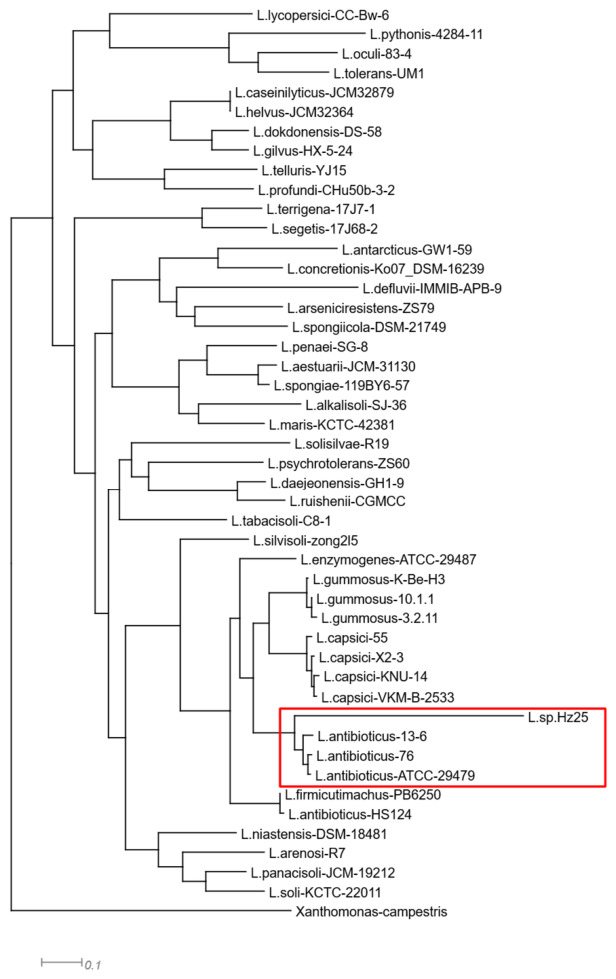
Phylogenetic tree of the strain Hz25 with closely related species of genus *Lysobacter*. The multilocus tree was built based on approximately 400 universal marker genes by PhyloPhlAn with the maximum-likelihood method. Strain Hz25 belongs to the specie *Lysobacter antibioticus*, which highlighted with the red contour.

To define the phylogenetic relationship of the strain Hz25 we used a traditional approach: construction of phylogenetic tree using PhyloPhlAn ver. 3.1.68 based on whole genome assemblies [25]. There are more than 90 known species belonging to the genus *Lysobacter* (https://lpsn.dsmz.de/genus/lysobacter, accessed on 31 March 2026). To clarify the tree, we retained only 47 closer species. The three closest strains of them (*L. antibioticus* 13-6, *L*. *antibioticus* 76, *L*. *antibioticus* ATCC 29479) belong to species *Lysobacter antibioticus* (Figure 1). These are well-known producers of antibacterial compounds [25].

**Figure 2:**
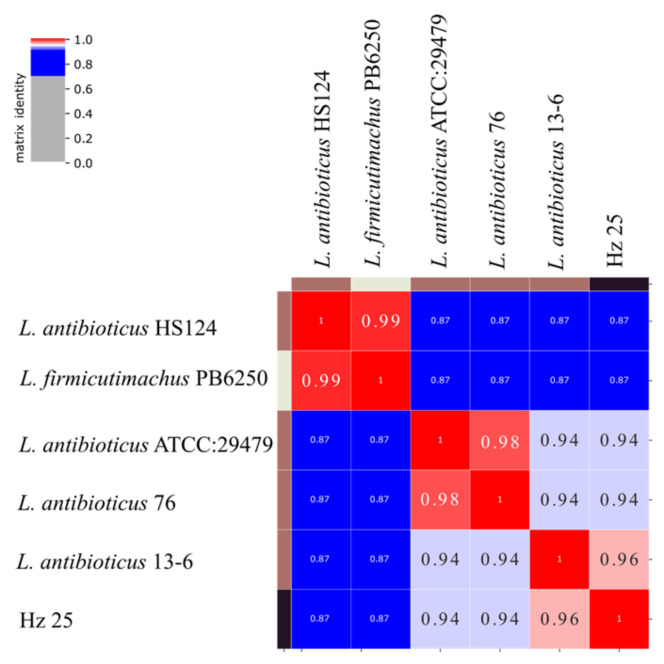
Heatmap of the average nucleotide identity (ANI) for *Lysobacter* sp. Hz25 and closely related strains. The scale indicates the percentage of sequence identity (from 0 to 1).

To define the phylogenetic relationship among the closest strains of *Lysobacter antibioticus*, we constructed a whole genome-based average nucleotide identity (ANI) distance matrix (presented as a heatmap) (Figure 2). This approach considers the entire genomic data and not limited to 16S rRNA diversity or 400 conservative genes used by PhyloPhlAn [26].

**Figure 3:**
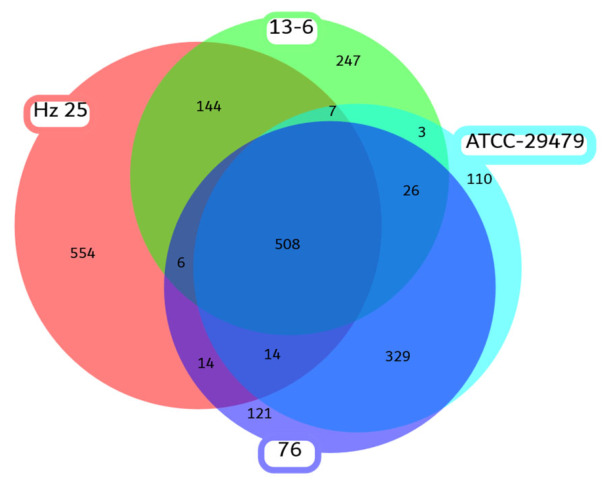
Venn diagram showing shared and unique gene clusters among Hz25 and closely related *L. antibioticus* strains (13-6, 76, and ATCC 29479).

A pan-genome analysis was performed to determine the strain-specific genes. The amino acid sequences from the whole genome of Hz25 were compared with three closely related strains of *Lysobacter antibioticus* (13-6, 76 and ATCC-29479) using Roary v. 3.13.0. This analysis revealed that, of the total 4936 predicted coding sequences (CDSs) in the Hz25 genome, 554 genes were unique to this strain (Figure 3). Functional annotation of these strain-specific genes highlighted several features that are potentially crucial for the Hz25 lifestyle and adaptability. We identified multiple components of pili apparatus (pilin, pseudopilin, PilO and PilW family protein), type IV secretion system proteins [27], and arsenic resistance system (*arsenate reductases*, *arsinothricin resistance N-acetyltransferase*, *arsenic resistance N-acetyltransferase*) [9]. A detailed description of motility-related genes is provided in Section 2.4.

### 2.3. Lytic Enzymes

Members of the genus *Lysobacter* employ various lytic enzymes to attack neighboring cells [2]. Strain Hz25 demonstrated chitinase activity: after seven days of incubation on minimal medium containing chitin, the strain formed pink colonies (2–3 mm in diameter), as reported briefly in [23]. Consistent with this phenotype, its genome harbors multiple chitinase-encoding genes, including three genes from family GH18 and four from family GH19 (annotation available at the CAZy DB: https://www.cazy.org/b36483.html, accessed on 1 October 2025). Amino acid sequence alignment of GH19 proteins with those from closely related strains (Supplementary Figure S1) revealed the presence of conserved catalytic domains and α-helices in *ChiC* and *ChiG*, consistent with those described by Yokomichi et al. [28].

Several bacteriolytic enzymes and one yeast-lytic enzyme have been identified in *L. capsici* XL1, including the bacteriolytic proteases L1, L4, L5; amidase L2; muramidase L3; metalloprotease Blp; and the yeast-lytic protease M4 [29]. Sequence comparison revealed that the Hz25 genome possesses four of these enzymes: *Blp* (73% identity, locus ACKN61_RS14770, M23 family metallopeptidase); serine protease *L1* (83%, ACKN61_RS16370, S1 family peptidase); metalloprotease *L4* (89%, ACKN61_RS10450, M35 family metallo-endopeptidase); and metalloprotease *M4* (88%, ACKN61_RS11830, M4 family metallopeptidase). Recently, the same research group discovered a new bacteriolytic amidase, *Ami*, produced by *L. capsici* XL1 and annotated as a Zn-dependent N-acetylmuramoyl-L-alanine amidase [30]. This protein is encoded in the Hz25 genome at locus ACKN61_RS21005, with 83% protein sequence identity to the reference sequence (accession no. WND81398).

### 2.4. Secretion Systems

The components of the type II secretion system (T2SS) are encoded by a gene cluster comprising *gspDEGM* and *xpsFHI* (loci ACKN61_RS05400 to ACKN61_RS05450). This cluster includes the cytoplasmic ATPase *GspE* and the major pseudopilin *GspG*.

**Figure 4:**
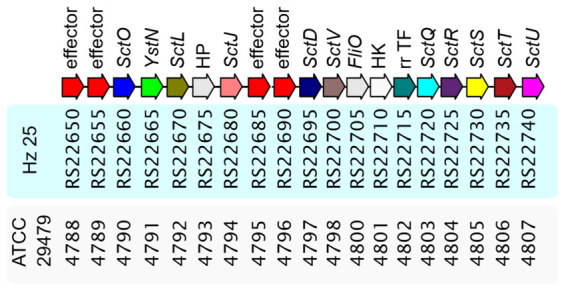
Genetic organization of the type III secretion system (T3SS) cluster in *Lysobacter* sp. Hz25 compared to the type strain *L. antibioticus* ATCC 29479. Arrows represent open reading frames (ORFs), with colors indicating functional categories: core structural components (e.g., *sctJ*, *sctL*, *sctO*, *sctQ*, *sctR*, *sctS*, *sctT*, *sctU*, *sctV*), ATPase (*yscN*), two-component system (HK, RR-TF), and putative effectors (RS22650, etc.). The secretin gene is located outside the main cluster (locus ACKN61_RS05450). ORFs of putative effectors for T3SS predicted by DeepSecE are marked in red.

The Hz25 genome also contains genes related to the type III secretion system (T3SS), including *sctDJLOQRSTUV* and *yscN*. A genetic map of the T3SS cluster, containing 17 ORFs in comparison with the type strain *L. antibioticus* ATCC 29479, is shown in Figure 4. A two-component system, comprising a serine histidine kinase (HK) and a response regulator transcription factor (RR-TF), was also annotated within the T3SS gene cluster. Several candidate effector proteins are encoded by genes in loci adjacent to the core T3SS genes (RS22650, RS22655, RS22685, and RS22690). Their functions were predicted using DeepSecE v 0.1.1 [31] with confidence scores ranging from 85% to 99%. The required secretin gene is located outside this region at locus ACKN61_RS05450 (the encoded protein, WP_408952643.1, contains the PF00263 family domain and the K02453 motif).

### 2.5. Motility

As demonstrated in our previous work, strain Hz25 is capable of gliding across a cell culture towards its prey using type IV pili [23]. The relevant gene clusters are distributed across several loci in the Hz25 genome. A cluster of five minor pilin genes (*pilEXWVT*) includes the anti-retraction protein gene *pilY1* (locus ACKN61_RS08145) and *fimT* (a pseudopilin). A separate cluster contains the *pilMNOPQ* gene complex (loci ACKN61_RS19575 to ACKN61_RS19595). Several additional genes involved in pilin synthesis, including *pilA*, *pilH*, *pilG*, *pilT*, *pilU*, and *gspG*, are located as single genes elsewhere in the genome. Notably, the Hz25 genome possesses the PilZ-PilB complex (loci ACKN61_RS05700, ACKN61_RS16490, and ACKN61_RS19720), which was recently described for *L. enzymogenes* OH11 [32].

### 2.6. Iron Acquisition and Secondary Metabolism

Iron uptake by strain Hz25 is mediated by the production of the siderophore lysochelin. Siderophore activity was confirmed on CAS medium in our previous work [23], where iron chelation resulted in a color change in the medium from blue to orange. Lysochelin is produced via the *lecABCDEF* gene cluster (loci ACKN61_RS12350 through ACKN61_RS12375), which has been previously described for *L. enzymogenes* [33].

As demonstrated in the case of *L. antibioticus* OH13 [17], the Hz25 genome contains four key genes associated with the biosynthesis of a compound structurally related to p-aminobenzoic acid (pABA). All four genes are present in the Hz25 genome: anthranilate synthase component I (*trpE*, locus ACKN61_RS04960), anthranilate synthase component II (ACKN61_RS04995), anthranilate phosphoribosyltransferase (*trpD*, ACKN61_RS05000), and aminodeoxychorismate synthase component I (*pabB*, ACKN61_RS24630). These genes have 97–100% sequence identity with the reference genes (accession numbers MG256384–MG256386).

In addition to siderophores, the Hz25 genome contains genes that encode pigments (xanthomonadin, aryl polyene) and antibacterial compounds (lysocin E, phenazine).

### 2.7. Calcium Homeostasis and Adaptation to Alkaline Soil Conditions

Two genes that are potentially involved in calcium tolerance were identified: locus ACKN61_RS12015, which encodes a 118-amino-acid protein containing an Excalibur calcium-binding domain; locus ACKN61_RS08540, which encodes a 337-amino-acid sodium/calcium antiporter.

## 3. Discussion

Interest in the genus *Lysobacter* has increased considerably in recent years, as evidenced by the wide range of studies devoted to its functions as a biocontrol agent, a plant growth promoter and an inducer of plant immunity [2]. However, descriptions of the genomic characteristics of *Lysobacter antibioticus* remain limited to a few studies [19,34,35,36]. To address this knowledge gap, we examined the genomic features of a recently isolated strain, *Lysobacter* sp. Hz25, whose genome has been deposited in NCBI GenBank (accession number CP175666) and which demonstrates a high BUSCO completeness score (98.9%). Phylogenetic analysis (see Methods for details) confirmed that *Lysobacter* sp. Hz25 belongs to the species *L. antibioticus*. The genome size of Hz25 (5.9 Mb) is typical for this species (genome sizes of reference strains: 5.1 Mb for HS124, 5.5 Mb for 13-6, 5.8 Mb for ATCC 29479, and 5.9 Mb for strain 76). We subsequently conducted a pan-genome analysis for these four closely related strains.

Despite its close taxonomic relationship with the aforementioned strains, Hz25 exhibits significant genomic plasticity. It possesses 554 unique genes (more than 10% of its genome) within the pan-genome of these closely related species. For comparison, strain 13-6 has 247 unique genes, ATCC 29479 has 110, and strain 76 has 121. The substantial number of unique genes in Hz25 may be attributable to specific environmental factors at its isolation site, including high insolation, cold winters and hot, dry summers, and alkaline, nutrient-poor soil.

The unique genes of strain Hz25 include those encoding pili components, proteins of the type IV secretion system (T4SS) [27], and an arsenic resistance system. Genes encoding type IV pili components are of particular interest. These include both unique genes (pilin, pseudopilin, *PilO*/*PilW*) and genes with high homology to those in related strains. A complete set of essential genes is present, including pilin genes; genes encoding enzymes responsible for pilus stabilization, assembly, function, regulation, and retraction; and genes of the *PilZ*–*PilB* complex, which is necessary for regulating pilus assembly [32]. The presence of these genes suggests that Hz25 can colonize soil particles using type IV pili-based motility and execute a micropredatory strategy, as proposed in previous studies [23].

The unique genes of Hz25 include those encoding proteins of the type IV secretion system (T4SS). In *Lysobacter*, T4SS has been previously described as a contact-dependent antagonistic system that facilitates attacks on target bacteria. The induction of antifungal activity in *Pseudomonas* via the delivery of non-toxic effectors through T4SS has also been documented [6,37]. The presence of unique variants of these systems in Hz25 may indicate a specific configuration of intermicrobial interactions that has evolved within the microbial community of the *Hedysarum zundukii* rhizosphere. Type IV pili-based motility, coupled with a type III secretion system, supports the colonization of plant roots and fungal hyphae and may confer competitive advantages to Hz25.

The Hz25 genome of contains several genes typically associated with arsenic resistance. These genes (*arsN2*, *arsC*, *arsR*) are located in close proximity to one another, although they do not form a canonical cluster. However, the *acr3* gene, which encodes an arsenite efflux pump [38], is absent from the genome. The presence of *arsC*, *arsR*, and *arsN2* in Hz25, together with the absence of *acr3* and *arsH*, suggests that these genes are unlikely to be involved in typical arsenic detoxification. This is consistent with the low arsenic content of the native soil (0.21 mg/kg).

Although *ars* operons are typically associated with bacteria from arsenic-contaminated environments [9], their presence in low-arsenic habitats is not uncommon and may reflect alternative physiological functions. Consistent with this hypothesis, each of these genes may contribute to processes beyond arsenic detoxification. For example, *ArsC* (arsenate reductase) belongs to a protein family that encompasses Spx-type transcription factors, which are involved in the disulfide stress response, a function that is conserved across various bacterial lineages. *ArsH* is an NADPH-dependent quinone reductase that protects against oxidative stress induced by menadione and other quinones [39]. *ArsR*, traditionally considered a repressor of the *ars* operon, has recently been demonstrated to regulate redox and metabolic networks globally even in the absence of arsenic [40].

The *arsN2* gene identified in Hz25 belongs to the GNAT family of N-acetyltransferases. Phylogenetic analysis revealed that the Hz25 *ArsN2* sequence is more closely related to glutamate N-acetyltransferases, which are involved in amino acid metabolism than to ArsN1-type enzymes. The latter mediate resistance to the antibiotic arsinothricin by acetylating its α-amino group [41]. This suggests that *ArsN2* in Hz25 may be involved in alternative acetylation reactions, potentially related to redox regulation or central metabolism, rather than serving solely in arsenic detoxification. It is therefore possible that the *ars* genes in Hz25 are not part of a functional detoxification pathway, but rather represent a conserved genetic repertoire with pleiotropic physiological roles.

In this context, the work of Luo et al. (2012) and Liu et al. (2015) on *L. arseniciresistens*, which was isolated from an iron ore mine in China, is particularly noteworthy [42,43]. Liu et al. identified arsenic resistance genes in the genome of *L. arseniciresistens* ZS79ᵀ and examined four additional *Lysobacter* strains (*L. concretionis* Ko07ᵀ, *L. daejeonensis* GH1-9ᵀ, *L. defluvii* IMMIB APB-9ᵀ, and *L. capsici* AZ78), all of which contained between one and five such genes. As members of the genus *Lysobacter* are frequently isolated from arsenic-rich environments [44,45], it is plausible that these genes are widespread and may have been maintained even in strains inhabiting low-arsenic niches due to their additional physiological functions.

Analysis of the genomes of the *L. antibioticus* strains most closely related to Hz25 (HS124, 13-6, 76, and ATCC 29479) revealed that strains 13-6, 76, and ATCC 29479 possess a compact *ars* cluster that includes *acr3*. However, strain HS124, like Hz25, lacks this efflux pump gene. Additionally, *L. daejeonensis* GH1-9ᵀ encodes only one *ars*-related gene, while *L. concretionis* Ko07ᵀ encodes two. These observations suggest that *ars* genes in *Lysobacter* may have functions other than arsenic resistance, and that their presence and organization vary considerably between strains.

In order to survive in the diverse rhizosphere environment described above, Hz25 employs a combination of features in its micropredatory strategy, including lytic enzymes and secretion systems. Many *Lysobacter* species exhibit antifungal activity. The GH18 (glycoside hydrolase family 18; 3 genes) and GH19 (glycoside hydrolase family 19; 4 genes) chitinases identified in the Hz25 genome provide the strain with a high capacity to hydrolyze both fungal and insect chitin. The yeast-lytic metallopeptidase M4 also contributes to the degradation of fungal cells.

Genomic analysis further demonstrated the ability of Hz25 to degrade bacterial cell walls. The bacteriolytic metallopeptidase Blp and metalloendopeptidase L4 (families M23 and M35, respectively) are required for the hydrolysis of bacterial cell wall peptides [29]. The functionality of these enzymes is complemented by amidase Ami (a Zn-dependent N-acetylmuramoyl-L-alanine amidase), which is highly homologous to the amidase from *L. capsici* XL1 described by Kudryakova et al. This amidase hydrolyzes the bond between the murein polysaccharide backbone and the peptide bridge [46]. The ability of this strain to degrade fungal and bacterial cells was confirmed experimentally [23].

While the presence of a type III secretion system (T3SS) in *L. enzymogenes* has been documented previously by de Bruijn in 2015 [47], this is the first detailed description of the complete cluster for *L. antibioticus*. In strain Hz25, the T3SS cluster exhibits a high degree of homology with that of the type strain ATCC 29479 and contains 17 open reading frames (ORFs), including conserved Sct components and a two-component regulatory system. The presence of four effector-encoding genes (predicted by DeepSecE [31] with 85–99% confidence score) located near the cluster suggests that the T3SS is functional. Notably, the secretin gene, which is necessary for the export of T3SS proteins, is located outside the main cluster at locus ACKN61_RS05450. This region also contains components of the type II secretion system (T2SS), which suggests potential coordination between the two secretion systems.

Although T3SSs are best known for their role in pathogenic bacteria, where they are used to inject effectors into host cells [48], they are increasingly recognized as important molecular machines for the interaction of non-pathogenic microorganisms with their eukaryotic hosts [49]. In soil, T3SSs are commonly found in bacteria that interact with fungi. There, they can mediate various ecological outcomes, including biocontrol, the facilitation of mycorrhization, and the migration along fungal hyphae [50]. In *Lysobacter*, the T3SS is known to be involved in interactions with fungal hyphae and bacterial cells, suggesting a possible role in biocontrol and micropredation [51]. However, as T3SS functions depend strongly on the ecological context, the presence of this system alone is insufficient to determine its specific role in the ecosystem [50]. We hypothesize that in Hz25, this system may mediate the delivery of effector proteins into target cells, potentially complementing the action of extracellular lytic enzymes. Alternatively, it may facilitate adhesion to fungal hyphae or plant surfaces, as has been demonstrated for other soil bacteria [49,50]. Elucidating the precise role of the T3SS in the ecology of Hz25 will require experimental validation, for example through targeted gene knockout.

The presence of the lysochelin biosynthesis gene cluster and experimentally confirmed siderophore activity in strain Hz25 is significant, given that iron acquisition is crucial for competition in the rhizosphere of *Hedysarum zundukii*. The carbolithozems of Cape Zunduk contain critically low levels of free iron (89 mg/kg). Furthermore, siderophore-mediated iron uptake may also protect Hz25 against oxidative stress [52].

In addition to lytic enzymes, the Hz25 genome encodes several antimicrobial compounds, including hypeptin, lysocin E, and a metabolite that is structurally similar to p-aminobenzoic acid (pABA). Hypeptin blocks bacterial cell wall biosynthesis and exhibits potent activity against a broad range of Gram-positive pathogens [53]. Lysocin E exerts bactericidal action by targeting menaquinone in the bacterial membrane, resulting in rapid cell lysis, including in pathogens such as *Staphylococcus aureus* and *Bacillus subtilis* [54]. Importantly, this antibiotic has demonstrated potent activity against *Mycobacterium tuberculosis*, including multidrug-resistant strains [55,56]. The urgent need for novel anti-tuberculosis agents is driven by the global burden of the disease, the emergence of multidrug-resistant strains, and the limited efficacy of existing treatment regimens [57]. Therefore, the presence of lysocin E biosynthetic genes in strain Hz25 is particularly noteworthy and warrants further investigation. p-Aminobenzoic acid is a key intermediate in folate synthesis, and its structural analog disrupts folate synthesis in target organisms. The production of this compound was described for *L. antibioticus* OH13 by Laborda et al. [17].

The synthesis of pigments (such as xanthomonadin and arylpolyenes, the genes for which were identified in Hz25) is known to be a mechanism of protection against UV radiation. This is particularly relevant in the climate of this region, which is characterized by extremely high insolation due to low rainfall. Furthermore, these pigments can protect cells from reactive oxygen species generated by natural geochemical processes in the soil or released by microorganisms for attack or defense.

The soil in which Hz25 is found has a high calcium content (>5000 mg/kg) and alkaline pH (8.5). In order to explore the genetic basis for adaptation to these conditions, we surveyed the Hz25 genome for genes involved in calcium homeostasis and alkaline stress tolerance.

The first gene, locus ACKN61_RS12015 (XLM94878), encodes a 118-amino-acid protein containing an Excalibur calcium-binding domain (Pfam PF04798), a motif associated with calcium binding that is found in secreted or membrane-associated proteins in various bacteria. The presence of a putative signal peptide (predicted by SignalP) suggests that this protein may be secreted into the periplasm or extracellular space, where it could potentially buffer extracellular Ca^2+^ and mitigate influx into the cell. The protein shares 95% identity with homologs in other *L. antibioticus* strains and 54–63% identity with Excalibur domain-containing proteins from diverse soil bacteria, including *Sphingomonas*, *Caulobacter*, and *Brevundimonas* species. This suggests that the protein plays a conserved role in environmental adaptation. The second gene, locus ACKN61_RS08540 (XLM96556), encodes a 337-amino-acid sodium/calcium antiporter (CaCA family, Pfam PF01699), a membrane protein predicted to export Ca^2+^ from the cytoplasm in exchange for Na^+^, thereby maintaining low intracellular calcium levels. The protein shows 99% identity with its counterpart in the type strain *L. antibioticus* ATCC 29479, and its homologs are widely distributed among *Lysobacter* and related genera, indicating a fundamental role in calcium homeostasis.

In conclusion, Hz25 has a significantly higher number of unique genes (554, comprising more than 10% of the pan-genome) than the three closest *L. antibioticus* strains, indicating significant genomic plasticity and suggesting adaptation to specific habitat conditions. For comparison, strain 13-6, which was isolated from the maize rhizosphere in China [19], has almost half as many unique genes (247). The unique genetic profile of Hz25 was likely shaped by its evolution in the Olkhon region of Lake Baikal, which is characterized by a sharply continental climate, low precipitation, and high insolation. It is also important to consider that *Hedysarum zundukii* grows on stony carbonate soils, which are characterized by alkaline pH (8.5), low iron availability, and high calcium and magnesium content.

Thus, the genome of the newly isolated Hz25 strain encodes an integrated genetic repertoire that supports micropredation, environmental adaptation, and competitiveness in the nutrient-poor rhizosphere. Its ability to acquire iron via siderophores, diverse antimicrobial and lytic properties, motility, and multiple secretion pathways suggest that Hz25 may contribute to the defense of *Hedysarum zundukii* against pathogens. By maintaining rhizosphere homeostasis, it may also facilitate the survival of this endemic species in the unique conditions of the Baikal region. In contrast to bacterial predators with a limited range of prey, the Hz25 genome encodes a wide variety of lytic enzymes, secretion systems, and contact-dependent antagonistic factors. This suggests the potential for Hz25 to interact with a broad range of bacterial competitors within soil microbial communities.

## 4. Materials and Methods

### 4.1. Bacterial Culture and Growth Media

The strain Hz25 was isolated from the rhizosphere of endemic plant *Hedysarum zundukii* Peschkova (Fabaceae), which is known for its drought resistance [58]. The soil at the isolation site (Cape Zunduk, Western Baikal region) is classified as a dark-humus carbolithosol (Russian soil classification), formed on carbonate rock eluvium under dry, stony steppe conditions [59]. The soil profile (AUca–BCca–Cca) is shallow (~26 cm), with high gravel content (>77% of particles > 1 mm) and a light texture (physical clay < 0.01 mm: 1.1%). Carbonates are abundant throughout the profile, as indicated by strong effervescence with HCl. The soil is sharply alkaline (pH_water_ 8.7) and contains high levels of organic carbon (12%) and total nitrogen (0.44%). Exchangeable calcium and magnesium are extremely high (>5000 mg/kg and >200 mg/kg, respectively), whereas available phosphorus (9 mg/kg) and available iron (89 mg/kg) are low. No contamination with heavy metals or petroleum products was detected [59]. Bacterial culture was cultivated on SYM medium (sucrose, 10 g/L; yeast extract, 5 g/L) containing agar (8 g/L) [24]. Libraries were prepared using PCR-free approach with Native Barcoding Kit 24 (Q20+), SQK-NBD112.24, (Oxford Nanopore, Oxford, UK). Long-read sequencing was performed on MinION with Flow Cell (R10.4.1), FLO-MIN114 (Oxford Nanopore Technologies, ONT, Oxford, UK). Basecalling was made with MinKNOW ver. 19.12.5.

### 4.2. Genome Assembly, Annotation and Phylogenetic Relationship

Merged ONT reads in FASTQ format were filtered with filtlong ver. 0.2.1 (parameters min_length = 1000, keep_percent = 90, https://github.com/rrwick/Filtlong, accessed on 1 December 2024). Illumina short reads were filtered with FastP ver. 0.23.4 (https://github.com/opengene/fastp, accessed on 1 December 2024) in default mode. Raw data are available via BioProject PRJNA1192387 (https://www.ncbi.nlm.nih.gov/bioproject/PRJNA1192387, accessed on 1 December 2024). Then the hybrid assembly was made by SPAdes ver. 3.13.1 with default parameters (https://github.com/ablab/spades, accessed on 1 December 2024). Genome completeness was evaluated by BUSCO ver. 5.8.0 on the final assembly using lineage database “xanthomonadales_odb10”, mode “genome” and other settings set to default (https://gitlab.com/ezlab/busco, accessed on 1 December 2024). Genome annotation was performed via PGAP (https://github.com/ncbi/pgap, accessed on 1 December 2024) during NCBI GenBank submission (genome acc. No CP175666 (https://ncbi.nlm.nih.gov/nuccore/CP175666), accessed on 1 December 2024). For automatic metabolic model construction, assembly for strain Hz25 was submitted to the RAST server [60]. Specific genes were selected from the subsystems associated with lytic activity, stress response, motility and bacterial competition; complete list is available in Table S2. The genes of interest were then analyzed manually along with literature-derived genes’ description.

For the phylogenetic analysis, 47 representative strains were selected to cover the taxonomic breadth of the genus *Lysobacter*, prioritizing those with complete or high-quality draft genomes available in NCBI GenBank at the time of analysis. A phylogenetic tree was built with PhyloPhlAn ver. 3.1.68 based on concatenated alignments of up to 400 conserved proteins using “supermatrix_aa” and low diversity mode with the “phylophlan” database [26].

For closely related strains, a whole-genome average nucleotide identity (ANI) distance matrix and a phylogenetic tree were generated using pyani v0.3.0 in ANIm mode (https://github.com/widdowquinn/pyani, accessed on 1 December 2025) with default settings and automatic fragmentation by pyani. These distances were calculated with MUMmer algorithm [61] using whole genomes divided into fragments as described in [62].

### 4.3. Comparative Genomics

To confirm the presence of the genes of interest we analyzed the genome assemblies of closely related strains: *L. antibioticus* 13-6, *L. antibioticus* 76 and *L. antibioticus* ATCC-29479 (NCBI GenBank accession numbers are specified in Table S1). For each gene (gene cluster), the pairwise and multiple-protein sequence alignments were analyzed to confirm the occurrence of the corresponding gene. As some loci were not annotated correctly in the Hz25 genome, so the conservative motif discovery via KOfam and Pfam databases was additionally used for functional annotation; the same approach was used previously [63] to predict functional domains. The pangenome analysis was performed using the Roary v. 3.13.0 with the default settings [64]. Clusters of secondary metabolite synthesis (pigments, antibiotics resistance) were predicted using the AntiSMASH ver. 8.0.4 online service [65]. Annotations of carbohydrate metabolism genes for the Hz25 genome of and closely related species were taken from the CAZy database [66].

### 4.4. Confirmation of Chitin Utilization

The ability of strain Hz25 to utilize chitin was previously demonstrated in brief communication [23]. Here, we provide a detailed description of the experimental protocol used in that study, as the method was only briefly outlined in the original report. To investigate the ability of Hz25 to utilize chitin as a carbon source, chitin was added to the minimal medium at a concentration of 1 g/L. The composition of the minimal medium was as follows (g/L): NH_4_Cl, 1; K_2_HPO_4_, 0.3; MgSO_4_·7H_2_O, 0.2; CaCl_2_·6H_2_O, 0.1. The pH was adjusted to 7.0. Agar was added to the medium at a concentration of 8 g/L to enable the bacterium to penetrate deeper and access the chitin that had settled to the bottom during solidification of the medium. The chitin-containing medium was poured as a thin upper layer over a base preventing desiccation layer of a medium containing [g/L] NaCl, 9; agar, 14. The use of a two-layer agar system for culturing *Lysobacter* on chitin was first described in [67].

Chitin was isolated from the hyphae of the fungus *Talaromyces pinophilus* using a modified version of the method described by Synowiecki & Al-Khateeb [68]. Briefly, the procedure was as follows. A fungal suspension, obtained by culturing in liquid SYM medium for 30 days, was centrifuged (10 min, 10,000 rpm). An equal volume of 10% NaOH was added to the pellet, and the mixture was sonicated for 1 h at 70 °C. After 24 h, the pellet was washed, resuspended in an equal volume of 10% HCl, and sonicated again with heating. The pellet was then washed to pH 7.0 and an equal volume of 3% H_2_O_2_ was added and the mixture was left for 24 h. The chitin was collected by centrifugation, dried in a silicone mold, and the resulting film was ground. The yield of chitin per liter of *T. pinophilus* suspension was 1–1.5 g.

### 4.5. Identification of Genes Involved in Calcium Homeostasis

Genes involved in calcium homeostasis were identified by searching the genome annotation for keywords (“calcium”, “Excalibur”, “antiporter”, “CaCA”), followed by manual verification of domain architecture using Pfam (PF04798 for the Excalibur domain; PF01699 for the CaCA family of antiporters).

## Figures and Tables

**Table 1:** Raw reads and genome feature statistics of *L.* sp. Hz25 genome.

Property	Value
Raw filtered reads (ONT), bp	4,179,714,568
Raw filtered reads (Illumina), bp	742,905,218
GC Content, %	66.94
Total CDS Count	4936
Genome size, bp	5,985,541
GenBank accession number	CP175666 (https://ncbi.nlm.nih.gov/nuccore/CP175666) accessed on 1 December 2024

## Data Availability

The whole genome sequence is available at NCBI GenBank acc. number CP175666 (https://ncbi.nlm.nih.gov/nuccore/CP175666 accessed on 1 December 2024) and raw data via BioProject PRJNA1192387 (https://www.ncbi.nlm.nih.gov/bioproject/PRJNA1192387 accessed on 1 December 2024).

## Supplementary Materials

The following supporting information can be downloaded at: https://www.mdpi.com/article/10.3390/ijms27093800/s1.
