# Supplementary material for: Genomic Features of the Micropredator Lysobacter sp. Hz25 Isolated from the Rhizosphere of Hedysarum zundukii
Source: Int J Mol Sci. 2026 Apr 24;27(9):3800. doi: 10.3390/ijms27093800 (PMC13163355; doi:10.3390/ijms27093800)
Supplement: Supplementary file 1 [file ijms-27-03800-s001.zip › Fig S1 Multiple alignments of catalytic domain of GH19 chitinase.pdf]

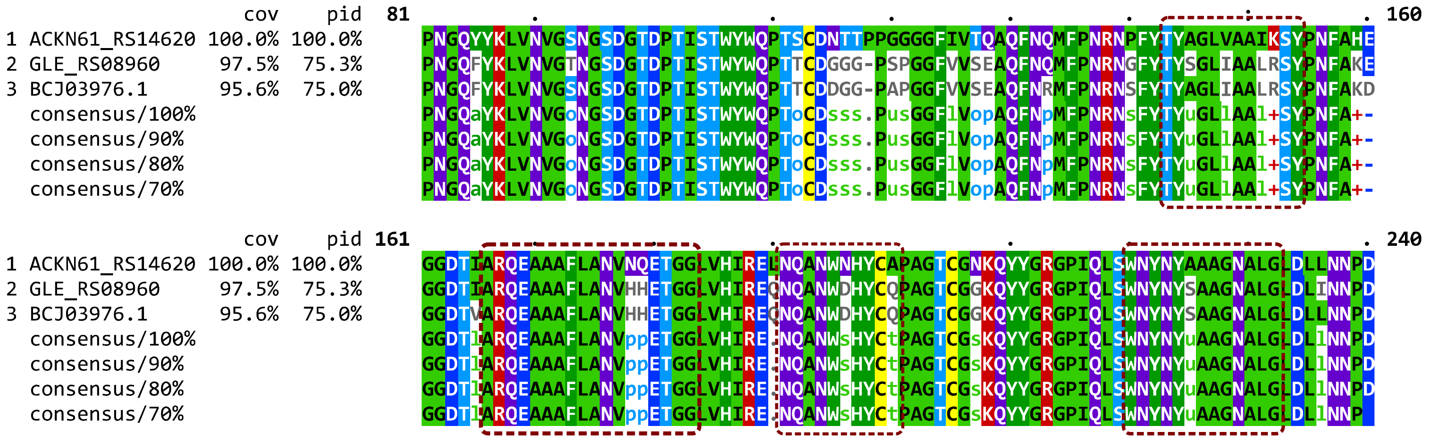

Figure S1. Multiple alignments of the catalytic domain of amino acid sequence of GH19 chitinase for the strains: *L. enzymogenes* C3 (locus GLE_RS08960), *Lysobacter* sp. MK9-1 (protein ID BCJ03976.1), *Lysobacter* sp. Hz25 (locus ACKN61_RS14620) The domain regions are shown with black dashed contours
